# CXCL13 is the major determinant for B cell recruitment to the CSF during neuroinflammation

**DOI:** 10.1186/1742-2094-9-93

**Published:** 2012-05-16

**Authors:** Markus C Kowarik, Sabine Cepok, Johann Sellner, Verena Grummel, Martin S Weber, Thomas Korn, Achim Berthele, Bernhard Hemmer

**Affiliations:** 1 Department of Neurology, Klinikum rechts der Isar, Technische Universität München, Ismaninger Str. 22, 81675, Munich, Germany

**Keywords:** CCL19, CCL21, CXCL12, CXCL13, BAFF, APRIL, B cells and plasmablasts

## Abstract

**Background:**

The chemokines and cytokines CXCL13, CXCL12, CCL19, CCL21, BAFF and APRIL are believed to play a role in the recruitment of B cells to the central nervous system (CNS) compartment during neuroinflammation. To determine which chemokines/cytokines show the strongest association with a humoral immune response in the cerebrospinal fluid (CSF), we measured their concentrations in the CSF and correlated them with immune cell subsets and antibody levels.

**Methods:**

Cytokine/chemokine concentrations were measured in CSF and serum by ELISA in patients with non-inflammatory neurological diseases (NIND, n = 20), clinically isolated syndrome (CIS, n = 30), multiple sclerosis (MS, n = 20), Lyme neuroborreliosis (LNB, n = 8) and patients with other inflammatory neurological diseases (OIND, n = 30). Albumin, IgG, IgA and IgM were measured by nephelometry. CSF immune cell subsets were determined by seven-color flow cytometry.

**Results:**

CXCL13 was significantly elevated in the CSF of all patient groups with inflammatory diseases. BAFF levels were significantly increased in patients with LNB and OIND. CXCL12 was significantly elevated in patients with LNB. B cells and plasmablasts were significantly elevated in the CSF of all patients with inflammatory diseases. CXCL13 showed the most consistent correlation with CSF B cells, plasmablasts and intrathecal Ig synthesis.

**Conclusions:**

CXCL13 seems to be the major determinant for B cell recruitment to the CNS compartment in different neuroinflammatory diseases. Thus, elevated CSF CXCL13 levels rather reflect a strong humoral immune response in the CNS compartment than being specific for a particular disease entity.

## Background

B cells play an essential role in the humoral immune response in neuroinflammation and serve as antigen presenting cells for T cells. Recruitment, clonal selection and expansion of B cells require a specialized milieu of secondary lymphoid organ chemokines and cytokines [1]. Chemokines, such as CCL19 (MIP-3β, Macrophage Inflammatory Protein-3), CCL21 (SLC, secondary lymphoid-tissue chemokine), CXCL12 (SDF-1, stromal cell-derived factor-1) and CXCL13 (BCA-1, B cell attracting chemokine-1), are known to influence migration of B cells [2]. These chemokines are constitutively expressed in lymphoid organs and regulate the migration and compartmentalization of lymphocytes and antigen presenting cells [3,4]. Some of the chemokines are present in the cerebrospinal fluid (CSF) and central nervous system (CNS), especially during neuroinflammation.

CXCL13 is produced by stromal cells, binds to the CXCR5 receptor and regulates homing of B cells and subsets of T cells to lymphoid follicles [5-7]. In addition, CXCL13 seems to play a role in the formation of ectopic lymphoid tissues within the CNS in chronic inflammatory CNS diseases [8,9]. Elevated CXCL13 levels were found in the CSF of patients with MS, neuroborreliosis and other inflammatory neurological diseases [4,10-13]. Furthermore, CXCL13 levels correlated with B and T cell numbers in the CSF and intrathecal immunoglobulin production [10]. CXCL13 has recently been suggested as a prognostic marker for multiple sclerosis and clinically isolated syndrome (CIS) [12-14]. Based on the observation that high CXCL13 levels are found in the CSF of patients with acute neuroborreliosis, CXCL13 was proposed as a specific diagnostic marker and a key regulator for B cells in acute Lyme neuroborreliosis [11,15-17].

CXCL12 binds to the receptor CXCR4 [18] and acts as a potent chemoattractant for B cells, plasma cells, T cells and monocytes [4,10]. The chemokine plays an important role in germinal center organization. CXCL12 is also expressed in the normal brain and is crucial for neuronal guidance [10,19]. Higher CSF levels of CXCL12 were found in inflammatory CNS diseases. CXCL12 was also found in MS lesions but CSF levels were only slightly elevated in these patients [1,10].

CCL19 and CCL21 bind to the CCR7 receptor, which is present on activated B cells, naive and central memory T cells, and dendritic cells. Both chemokines strongly guide subsets of T cells, B cells and mature dendritic cells into secondary lymphatic organs [1,20,21]. Additionally, CCL19 levels have been shown to be increased in the CSF of patients with inflammatory neurological diseases and MS [1,22]. CCL19 mRNA was shown to be overexpressed in MS lesions [4,22].

The cytokines BAFF (B cell activating factor) and APRIL (A Proliferation Inducing Ligand) are members of the TNF (Tumor Necrosis Factor) family and are both expressed by monocytes, macrophages, dendritic cells, astrocytes and, at lower levels, by T cells [23]. APRIL binds to the receptors BCMA (B cell maturation), TACI (transmembrane activator and CAML interacting protein) and syndecan-1 (CD138), BAFF receptors comprise BAFF-R, TACI and BCMA. These receptors are mainly expressed on B cells and to a lower extent on T cells [23,24]. APRIL and BAFF are key factors for the development and survival of B cells; furthermore, BAFF acts as a potent B cell activator [25]. It has been shown that BAFF mRNA is up-regulated in MS lesions and secreted by astrocytes upon stimulation [25]. APRIL protein expression was up-regulated in astrocytes in MS patients whereas APRIL protein levels were not elevated in the CSF [26-28].

Taken together, these data suggest a possible role for each of these chemokines and cytokines in neuroinflammation. So far, most cytokines and chemokines were studied in small cohorts of patients often focusing on a single specific neuroinflammatory disease.

To obtain a more complete picture of these cytokines and chemokines, we performed a study in a set of 108 patients with non-inflammatory neurological diseases (NIND), clinically isolated syndrome (CIS), multiple sclerosis (MS), Lyme neuroborreliosis (LNB) and patients with other inflammatory neurological diseases (OIND). The objective of the present study was to determine which cytokines and chemokines are most strongly associated with the occurrence of B cells, plasmablasts and the secretion of antibodies in the CSF compartment, irrespective of the specific disease. In parallel to measurements of chemokine and cytokine concentrations by ELISA, seven-color flow cytometry was performed in 107 patients to dissect the major immune cell subsets in the CSF.

## Methods

### Patients

Patients were recruited at the Department of Neurology of the Technische Universität München. All CSF samples were primarily obtained for routine diagnostic work-up, patients consented to the scientific use of their biosamples. Furthermore, the ethics committee of the Technische Universität München approved the scientific use of CSF biosamples.

A total of 20 patients with NIND, 30 patients with CIS, 20 patients with relapsing remitting MS, 8 patients with LNB, and 30 patients with OIND were included in this study. Patients with non-inflammatory neurological diseases suffered from headache (6), hypesthesia of unknown origin/somatoform disorders (7), Bell’s palsy (2, LNB and herpes virus infection were excluded), ischemic optic neuropathy/stroke (2), epileptic seizure (1), normal pressure hydrocephalus (1) and pseudotumor cerebri (1). Patients with other inflammatory neurological diseases had the following diagnoses: autoimmune cerebellitis (1), sarcoidosis (1), bacterial meningoencephalitis (3, Campylobacter fetus, Haemophilus influencae, Listeria monocytogenes), viral meningoencephalitis (3, herpes virus), meningoencephalitis of unknown origin (6) and HIV (16). HIV patients suffered from meningoencephalitis (3, unknown pathogen), meningoradiculitis (1), myelitis of unknown origin (1), syphilis (3) and unspecific neurological symptoms (8). Concerning HIV therapy, six patients received an antiretroviral therapy, six patients did not receive an antiretroviral therapy. No information on treatment was available for four HIV patients. None of the patients had received any immunomodulatory drugs before a spinal tap was performed. Two MS patients were treated with steroids before a spinal tap was performed. All other patients were steroid naïve at the time of spinal tapping. Further details are displayed in Table 1.

**Table 1:** Demographic data and CSF parameters

	**NIND**	**CIS**	**MS**	**LNB**	**OIND**
**Demographic data**					
Samples	20	30	20	8	30
Samples for Immuno-phenotyping CSF/Blood	19/13	30/28	20/15	8/3	30/24
Age (years)	32 (18 to 77)	31 (17 to 51)	38 (18 to 69)	57 (37 to 78)	41 (18 to 73)
Sex female/male	10/10	15/15	11/9	6/2	11/19
Phase of disease (Number of patients)	Acute^a^ symptoms (20)	Acute^a^ relapse (27), remission (3)	Acute^a^ relapse (15), remission (5)	Acute^a^ (6), chronic^b^ (2)	Acute^a^ (22), remission (2), unknown (6)
**CSF parametes**					
CSF cell count per μl	2 (1 to 5)	7 (1 to 29)	7 (0 to 81)	106 (9 to 280)	33 (2 to 512)
Q_Albumin_	5 (3.0 to 9.6)	5 (3.1 to 10.1)	5 (3.3 to 7.5)	12 (6.0 to 25.9)	8 (3.7 to 55.5)
Intrathecal IgG production	0/20	17/30	13/18	7/8	10/29
Intrathecal IgA production	0/20	0/30	2/18	5/8	9/29
Intrathecal IgM production	0/20	4/30	5/18	6/8	6/29
OCBs (+/-)	0/20	(25/0)^c^	(19/1)	(6/2)	(13/17)
Impairment blood-CSF barrier	2/18	8/30	3/20	6/8	21/30

^a^ Acute: onset of symptoms within four weeks.

^b^ Chronic: duration of symptoms for at least six months.

^c^ In five patients a single band in the CSF was observed.

Abbreviations: *CIS* clinically isolated syndrome, *CSF* cerebrospinal fluid, *LNB* Lyme neuroborreliosis, *MS* multiple sclerosis, *NIND* non-inflammatory neurological diseases, *OCBs* oligoclonal bands, *OIND* patients with other inflammatory neurological diseases; *+/−* sample positive/negative.

### Specimen handling and routine CSF testing

A total of 5 to 15 ml CSF was obtained by lumbar spinal tap with an atraumatic needle. Ten milliliters of EDTA blood were drawn for immunophenotyping and 10 ml of whole blood for serum analyses of albumin and IgG. CSF and blood from each patient were always drawn on the same occasion (a maximum of 10 minutes apart). Samples were processed according to the BioMS guidelines and stored at −80°C [29]. The average time between sample collection and freezing was 57 minutes (minimum 15 minutes, maximum 2 hours 25 minutes).

For routine CSF workup, CSF mononuclear cells were immediately (that is, within 30 minutes after the spinal tap) counted in a Fuchs Rosenthal chamber (Roth, Karlsruhe, Germany). Total protein, albumine, IgG, IgM and IgA concentrations in CSF and serum were determined by nephelometry (Siemens ProSpec®, Eschborn, Germany, according to the manufacturer`s instructions) and oligoclonal bands were investigated by isoelectric focusing followed by silver staining. Qalb, QIgG, QIgA and QIgM were calculated as described before [30].

### ELISA

All ELISA Kits were evaluated in duplicates and showed a variability of 2.2 to 5.3% for the same samples (APRIL ELISA: 5.3% (average deviation from the mean), BAFF ELISA: 2.9% (average deviation from the mean), CXCL12 ELISA: 4.7% (average deviation from the mean), CXCL13 ELISA: 4.5% (average deviation from the mean), CCL19 ELISA: 2.2% (average deviation from the mean) and CCL21 ELISA: 4.8% (average deviation from the mean)).

We pretested more than 100 samples for assay variability and consistency before we conducted the final experiment with all samples at the same time. Given the limitation in CSF sample volume, the need to avoid thawing and refreezing of samples and the aim to keep the assay times as short as possible (that is, avoid lengthy washing and processing times), we performed the final experiments with single wells in order to allow measurement of all serum and CSF samples in the same ELISA assays at the same time. Nunc-ImmunoMaxisorb 96-well immunoplates (Nunc, Roskilde, Denmark) were coated with 100 μL of 2 μg/ml CXCL13 capture antibody (DuoSet R&D, Minneapolis, MN, USA), 2 μg/ml CXCL12 capture antibody (DuoSet R&D), 0.8 μg/ml CCL19 capture antibody (DuoSet R&D,), 4 μg/ml CCL21 capture antibody (DuoSet R&D), 0.5 μg/ml APRIL capture antibody (Bender, Vienna, Austria) or 1 μg/ml BAFF capture antibody (Antigenix, Huntington Station, NY, USA) in phosphate-buffered saline (PBS) (PAA, Pasching, Austria) at 4°C overnight. Plates were washed with PBS/0.05% Tween-20 (PBST) (Sigma-Aldrich, St. Louis, MO, USA) and nonspecific binding sites were blocked with 200 μL of 1% bovine serum albumin (BSA) (Sigma-Aldrich, St. Louis, MO, USA) in PBST for 2 h. After three washing cycles, 100 μL of undiluted CSF were added for 2 h at room temperature (RT). Plates were washed three times with PBST and incubated with 100 μl of respective biotinylated detection antibody according to the manufacturer’s instructions. Plates were washed three times with PBST and were incubated with streptavidin-horseradish-peroxidase conjugate at RT. Reaction was stopped after 10 to 15 minutes and absorbance was read at 405 nm. Serial dilutions of respective proteins were used as standards and standard curves were generated to determine protein concentrations.

### Immunophenotyping

Flow cytometric analysis of immune cell subsets was performed as described previously [31]. Fresh CSF was immediately spun down at 300 G for 10 minutes. The supernatant was removed and the pellet resuspended in 180 μl phosphate-buffered saline (PBS) (PAA, Pasching, Austria) with 2% fetal calf serum (FCS) (Invitrogen, Darmstadt, Germany). A minimum number of 6,000 to 10,000 cells (yielding 2,500 cells per analysis) were used for the staining with a volume of 30 μl in each well of a 96-well plate (Nunc, Roskilde, Denmark). For cell staining, the pellet was resuspended in 15 μl antibody solution and incubated for 20 minutes at 4°C. After a washing step with 180 μl (PBS) supplemented with 2% (FCS), the pellet was resuspended in 180 μl of PBS wash solution for flow cytometric analysis (Beckman Coulter Cyan, Brea, CA, USA).

The following monoclonal antibodies were used for staining: CD4 PerCP, CD3 APC-Cy7, CD45 VM (all BD Bioscience, Bedford, MA, USA), CD19 ECD, CD56 APC, CD14 FITC and CD138 PE (all Beckman Coulter). This allowed to differentiate CD4^+^ T cells (CD45^+^ CD3^+^ CD4^+^), CD8^+^ T cells (CD45^+^ CD3^+^ CD8^+^), monocytes (CD45^+^ CD14^+^), NK cells (CD45^+^ CD56^+^), B cells (CD45^+^ CD19^+^ CD138^-^) and plasmablasts (CD45^+^ CD19^+^ CD138).

### Statistical analysis

The data were analyzed with the Kolmogorov-Smirnov test and did not show a Gaussian distribution. Therefore, the Kruskal-Wallis test with Bonferroni correction for multiple testing (Dunn`s procedure) was applied to test for significant differences among the different patient cohorts. Values of *P* <0.05 were considered to be significant. In order to test for correlations between the different parameters, Spearman test was applied. Furthermore, we applied stepwise multiple regression analysis.

## Results

### Cytokine levels in the CSF

**Figure 1:**
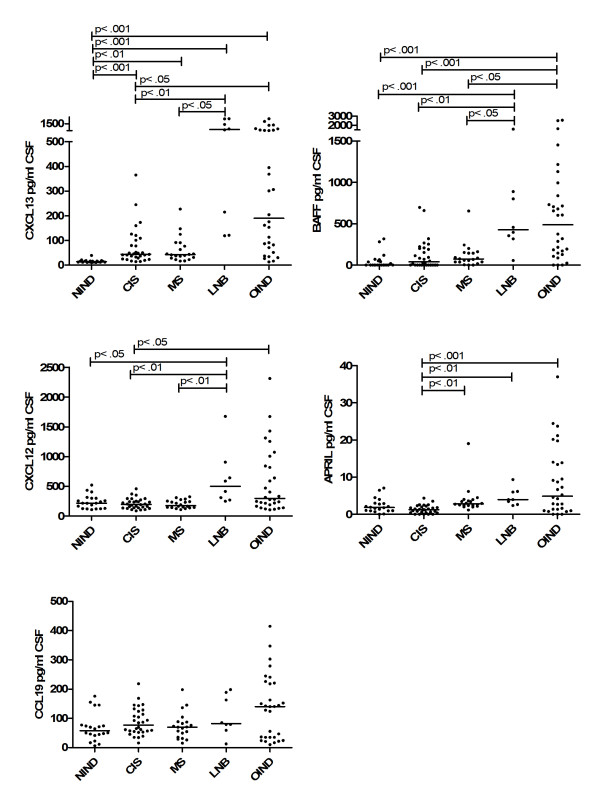
** Chemokine/Cytokine levels in CSF.** CXCL13, CXCL12, CCL19, BAFF and APRIL CSF levels of patients with non-inflammatory neurological diseases (NIND), clinically isolated syndrome (CIS), multiple sclerosis (MS), Lyme neuroborreliosis (LNB) and patients with other inflammatory neurological diseases (OIND) are shown. CCL21 was not detectable in the CSF. Horizontal line indicates median, significant *P*-values for the comparison among groups are displayed.

We determined the CSF cytokine and chemokine levels in NIND, OIND, CIS, MS and LNB patients (Figure 1). The most pronounced effects were observed for CXCL13. Patients with CIS, MS, Lyme disease and OIND all showed higher CSF CXCL13 levels than patients with NIND (*P* <0.001, *P* <0.01, *P* <0.001 and *P* <0.001 respectively). The highest concentrations were found in LNB patients, who had significantly higher levels than patients with CIS or MS patients (*P* <0.01 and *P* <0.05) but not OIND patients. Patients with OIND and LNB showed a heterogenous expression and both patient groups displayed a subpopulation with very high values. In the subgroup of OIND patients, highest CSF CXCL13 levels were similar to those observed in patients with acute Lyme disease. The latter OIND patients comprised HIV infections (5), bacterial meningoencephalitis (2), severe viral encephalitis (1), meningoencephalitis of unknown origin (1) and neurosarkoidosis (1). These findings are largely in line with previous studies, which have also reported elevated CSF levels in patients with MS, CIS, LNB and other inflammatory diseases [10,12,14].

CXCL12 was found in all CSF samples (Figure 1). Patients with LNB showed the highest levels, which were higher than those of patients with NIND, CIS and MS (*P* <0.05, *P* <0.01 and *P* <0.01). Patients with OIND had higher values than patients with CIS (*P* <0.05). We found no differences between patients with NIND, CIS and MS, which is in line with two previous studies [1,14]. Only one other study found significantly higher CXCL12 concentrations in MS patients [10].

CCL19 was also detectable in all CSF samples (Figure 1). Patients with CIS, MS, OIND and LNB showed slightly higher levels than patients with NIND but the differences failed to reach significance. This contrasts with a previous study which found significantly higher CCL19 values during inflammatory diseases such as MS and OIND [1].

BAFF was detectable in the CSF of most patients except 10 with NIND, 12 with CIS, 2 with MS and 3 with OIND (Figure 1). The highest BAFF concentrations were found in patients with LNB and OIND, where levels were significantly higher than those in patients with NIND, CIS and MS (*P* <0.001, *P* <0.01, and *P* <0.05 in LNB and resp. *P* <0.001, *P* <0.001 and *P* <0.05 in OIND). CSF levels of MS and CIS patients were also slightly elevated compared to patients with NIND, but the differences failed to reach statistical significance. This is in line with a previous study that could not find significant differences among CIS, MS and NIND patients [26].

APRIL was detectable in all CSF samples except one with NIND, five with CIS and two with OIND. Patients with CIS showed a homogeneous expression, which was significantly lower than those found in patients with MS, LNB and OIND (*P* <0.01, *P* <0.01 and *P* < .001). There were no significant differences among the other groups. In previous studies, no significant differences were observed among patients with MS, CIS and NIND [27,28].

CCL21 was not detectable in the CSF as reported before [22].

In serum, only minor differences among the patient groups were observed (Additional file 1: Figure S1). CXCL13 was higher in OIND patients compared to NIND and MS patients (*P* <0.01, *P* <0.05). BAFF serum concentrations were higher in LNB and MS than in NIND patients (*P* <0.05, *P* <0.05). APRIL levels were higher in the serum of LNB than CIS patients (*P* <0.05).

All patients with impaired blood-CSF barriers (defined as albumin quotient ((Albumin_CSF_/ Albumin_Serum_) x 10^-3^) ≥8.0) showed higher chemokine/cytokine levels in CSF than patients with intact barriers. We found no evidence that gender impacts on the level of chemokine/cytokine levels in serum or CSF. We also did not observe a major impact on age of the patient and the relevant levels of cytokine/chemokines in blood and CSF.

### Immune cell subsets within the CSF of different patient cohorts

**Figure 2:**
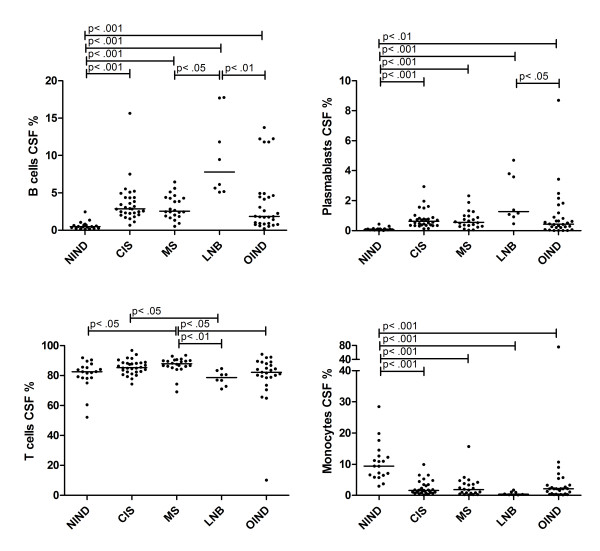
** Immune cell subsets in CSF.** The percentage of B cells, plasmablasts, T cells and monocytes in the CSF of patients with NIND, CIS, MS, LNB and OIND are shown. Values are given as the proportion of flow cell count in the CSF, the horizontal line indicates median. Significant *P*-values for the comparison among groups are displayed.

In all CSF samples, immune cell phenotyping was performed and the distribution compared among the different patient groups (Figure 2). The fraction of B cells was elevated in patients with CIS, MS, LNB and OIND when compared to patients with NIND (*P* <0.001 for all four patient cohorts). Patients with LNB displayed the highest values, the difference reached significance when compared to patients with MS and OIND (*P* <0.05 and *P* <0.01).

Also the fraction of plasmablasts was elevated in patients with CIS, MS, LNB and OIND compared to patients with NIND (*P* <0.001, *P* <0.001, *P* <0.001 and *P* <0.01). Again, patients with LNB showed the highest median values which were higher than in patients with OIND (*P* <0.05).

Concerning T cells, MS patients had slightly elevated fractions compared to patients with NIND (*P* <0.05), LNB (*P* <0.01) and OIND (*P* <0.05). Also patients with CIS displayed higher values than patients with LNB (*P* <0.05).

Regarding monocytes, patients with CIS, MS, LNB and OIND had lower values than patients with NIND (*P* <0.001 for all four patient cohorts).

### Correlation among chemokine/cytokine levels, CSF parameters and immune cell subsets

CSF levels of CXCL13, CXCL12, CCL19, BAFF and APRIL were compared to CSF parameters, the proportion of all CD19^+^ B cells, CD19^+^CD138^-^ B cells, CD19^+^CD138^+^ plasmablasts, CD3^+^ T cells and CD14^+^ monocytes.

The concentration of all cytokines/chemokines significantly correlated with CSF cell count and albumin quotient (Table 2). CXCL13 showed the lowest *P* and highest r values (*P* <0.0001, r = 0.7583, resp. *P* <0.0001, r = 0.5066). Concerning the intrathecal IgG synthesis, only CXCL13 correlated with the extent of intrathecal IgG synthesis (*P* <0.0002, r = 0.3540). APRIL, BAFF, CXCL12 and, especially, CXCL13 levels (*P* <0.0001, r = 0.3965) in CSF significantly correlated with the extent of intrathecal IgA synthesis. APRIL, BAFF and CXCL13 levels (*P* = 0.0002, r = 0.3567) in CSF correlated with intrathecal IgM synthesis. CXCL13 showed the lowest *P* and highest r values for all correlations with IgG, IgA and IgM. Only CXCL13 showed a significant correlation with the presence of oligoclonal bands (OCBs) in the CSF (*P* = 0.0104, r = 0.2539).

**Table 2:** Correlation of CSF cytokine/chemokine levels and CSF parameters

	**APRIL**	**BAFF**	**CXCL12**	**CXCL13**	**CCL19**
Cell count	***P <0.0001***	***P <0.0001***	***P <0.0001***	***P <0.0001***	***P <0.0001***
	***r = 0.4350***	***r = 0.4967***	***r = 0.4954***	***r = 0.7583***	***r = 0.5461***
Q_alb_	***P = 0.0067***	***P = 0.0003***	***P <0.0001***	***P <0.0001***	***P <0.0001***
	***r = 0.2669***	***r = 0.3507***	***r = 0.5011***	***r = 0.5066***	***r = 0.4600***
Intrathecal IgG	*P* = 0.9332	*P* = 0.2370	*P* = 0.8147	***P = 0.0002***	*P* = 0.0808
	r = 0.008406	r = 0.1175	r = -0.02337	***r = 0.3540***	r = 0.1737
Intrathecal IgA	***P = 0.0305***	***P = 0.0034***	***P = 0.0060***	***P <0.0001***	*P* = 0.2992
	***r = 0.2143***	***r = 0.2863***	***r = 0.2692***	***r = 0.3965***	r = 0.1038
Intrathecal IgM	***P = 0.0018***	***P = 0.0330***	*P* = 0.0540	***P = 0.0002***	*P* = 0.5006
	***r = 0.3049***	***r = 0.2103***	r = 0.1904	***r = 0.3567***	r = 0.06745
OCBs	*P* = 0.5013	*P* = 0.8673	*P* = 0.0971	***P = 0.0104***	*P* = 0.8844
	r = -0.06767	r = 0.01684	r = -0.1660	***r = 0.2539***	r = 0.01465
All B cells	*P* = 0.4474	***P = 0.0478***	***P = 0.0312***	***P <0.0001***	*P* = 0.0855
(CD19^+^)	r = 0.07422	***r = 0.1918***	***r = 0.2084***	***r = 0.6264***	r = 0.1670
B cells	*P* = 0.8110	*P* = 0.0945	*P* = 0.0859	***P <0.0001***	*P* = 0.2317
(CD19^+^D138^-^)	r = 0.02339	r = 0.1625	r = 0.1668	***r = 0.5756***	r = 0.1166
Plasmablasts	***P = 0.0317***	***P = 0.0100***	***P = 0.0426***	***P <0.0001***	***P = 0.0067***
(CD19^+^CD138^+^)	***r = 0.2078***	***r = 0.2481***	***r = 0.1965***	***r = 0.5828***	***r = 0.2605***
T cells	*P* = 0.7699	*P* = 0.1733	***P = 0.0036***	*P* = 0.5173	***P = 0.0337***
(CD3^+^)	r = -0.02931	r = -0.1359	***r = -0.2861***	r = -0.06485	***r = -0.2105***
Monocytes	***P = 0.0050***	***P <0.0001***	***P = 0.0034***	***P <0.0001***	***P = 0.0030***
(CD14^+^)	***r = -0.2706***	***r = -0.3866***	***r = -0.2819***	***r = -0.5834***	***r = -0.2861***

Spearman test was applied to correlate CSF cytokine/chemokine levels with CSF parameters or percentage of immune cells. All samples (with and without an intact blood-CSF barrier) were included in the analysis, significant values are displayed in bold. Abbreviations: *CSF* cerebrospinal fluid, *Q*_*alb*_ albumin quotient, *OCBs* oligoclonal bands.

**Figure 3:**
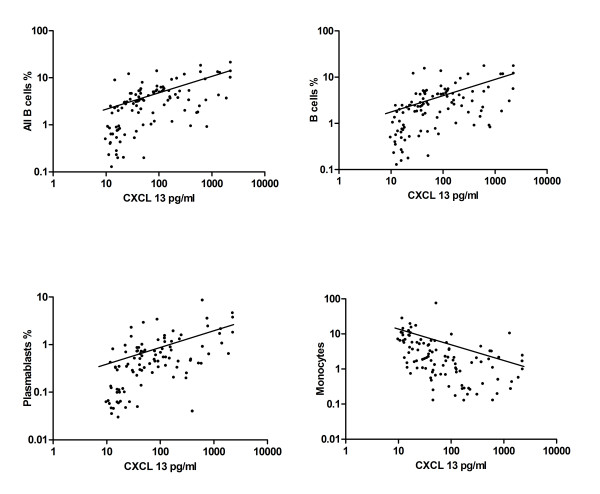
** Correlation analysis.** Correlations among CXCL13 CSF levels and CSF cell subsets (all B cells (CD19^+^), B cells (CD19^+^CD138^-^), plasmablasts (CD19^+^CD138^+^) and monocytes (CD14^+^)) are shown. The *P*-values for the correlation analysis are given in Table 2.

The proportion of all CD19^+^ B cells correlated with CSF levels of BAFF (*P* = 0.0478, r = 0.1918), CXCL12 (*P* = 0.0312, r = 0.2084) and CXCL13 (*P* <0.0001, r = 0.6264). CD19^+^CD138^-^ B cells only correlated with CXCL13 (*P* <0.0001, r = 0.5756). CD19^+^CD138^+^ plasmablasts correlated with APRIL (*P* = 0.0317, r = 0.2078), BAFF (*P* = 0.01, r = 0.2481), CXCL12 (*P* = 0.0426, r = 0.1965), CCL19 (*P* = 0.0067, r = 0.2605) and CXCL13 (*P* <0.0001, r = 0.5828) CSF levels. The proportion of T cells inversely correlated with CXCL12 (*P* = 0.0036, r = −0.2861) and CCL19 (*P* = 0.0337, r = −0.2105). A negative correlation was observed between all cytokine/chemokine CSF levels and the proportion of monocytes in CSF; CXCL13 again showed the strongest correlation (*P* <0.0001, r = −0.5834) (Figure 3, Table 2).

In patients with intact blood CSF barrier, only the chemokines CXCL12, CCL19, and most pronounced CXCL13 correlated with CSF cell count (*P* <0.0001, r = 0.5978). Intrathecal IgG synthesis correlated with CSF BAFF, CCL19 and CXCL13 levels (*P* <0.0001, r = 0.5445). Only CXCL13 correlated with intrathecal IgA synthesis (*P* = 0.0130, r = 0.3089). Intrathecal IgM synthesis significantly correlated with APRIL and CXCL13 levels (*P* = 0.0109, r = 0.3164). Again, only CXCL13 significantly correlated with oligoclonal bands (*P* = 0.0109, r = 0.3089). CXCL13 CSF levels correlated with all CD19^+^ B cells (*P* <0.0001, r = 0.666), CD19^+^CD138^-^ B cells (*P* <0.0001, r = 0.6889), CD19^+^CD138^+^ plasmablasts (*P* <0.0001, r = 0.5818) and inversely with monocytes (*P* >0.0001, r = −0.5980) in patients with intact blood-CSF barrier. Also CCL19 significantly correlated with B cells, plasmablasts and inversely with monocytes. Again, CXCL13 showed lowest *P* and highest r values for correlations with IgG, IgA, IgM, B cells and plasmablasts (Additional file 2: Table S1).

Correlation analysis was also performed with absolute cell counts of the different immune cell subsets again suggesting a major role for CXCL13 for the recruitment of B cells (Additional file 3: Table S2).

Further correlation subanalyses of the quotient Q_CSF/Serum_ (quotient CSF cytokine/chemokine and serum cytokine/chemokine) and B cells revealed that only the Q_CSF/Serum_ of CXCL13 significantly correlated with CD19^+^ B cells (*P* <0.0001, r = 0.4049), CD19^+^CD138^-^ B cells (*P* = 0.0002, r = 0.3698) and CD19^+^CD138^+^ plasmablasts (*P* = 0.0007, r = 0.3392).

Levels of APRIL (*P* = 0.0003, r = 0.3559), BAFF (*P* = 0.0048, r = 0.2796) and CXCL13 (*P* <0.0001, r = 0.3825) correlated between CSF and serum. In samples with an intact blood-CSF barrier APRIL (*P* = 0.0250, r = 0.3048) and CXCL13 (*P* = 0.0018, r = 0.4018) CSF values significantly correlated with serum values.

In samples with an intact blood-CSF barrier, we observed no correlation among the concentrations of the different cytokines/chemokines in the CSF. In contrast, samples with a disrupted blood-CSF barrier showed a significant correlation among all cytokines/chemokines.

### Stepwise multiple regression

In order to further clarify the impact of the different chemokines on the CSF cell subpopulations, we performed stepwise multiple regression. All chemokines/cytokines were introduced into the regression model and in a first step separately tested for a correlation with the different immune cell subsets. Thereby the chemokines/cytokines had to fulfill the entry criteria of *P* <0.05. In a second step, the included chemokines/cytokines were tested in parallel to evaluate the factor with the highest predictive potency for the presence of a specific immune cell subset in the CSF.

For CSF B cells, CXCL13, CXCL12 and BAFF were included into the regression model as predictive factors and CXCL13 turned out to be the most predictive factor. CXCL13 and BAFF were included in the regression model for plasmablasts with CXCL13 being the most predictive factor. Concerning CSF T cells, BAFF and APRIL were included in the statistical model, and BAFF showed a stronger correlation with T cells than APRIL. For monocytes no chemokines/cytokines passed the entry criteria for the regression model (Table 3).

**Table 3:** Stepwise multiple regression

	**Stepwise multiple regression**				**Predictor variables**		
	**Model**				***Variable***	***Beta***	***P***
	***F***	***df***	***P***	**R**^**2**^			
**B cells all**	21.650	3	0.000	0.391	CXCL13	0.745	0.000
					CXCL12	−0.490	0.001
					BAFF	0.320	0.012
**B cells**	18.098	2	0.000	0.262	CXCL13	0.616	0.000
					CXCL12	−0.271	0.010
**Plasmablasts**	20.265	2	0.000	0.287	CXCL13	0.413	0.000
					BAFF	0.234	0.009
**T cells**	6.001	2	0.003	0.110	BAFF	−0.498	0.001
					APRIL	0.296	0.05
**Monocytes**					No variables included		

Stepwise multiple regression was applied to determine the most predictable cytokine/ chemokine for the presence of certain immune cells in the CSF. Variables (cytokines/ chemokines), which fulfilled the entry criteria of *P* <0.05 for the regression model, are displayed. For B cells and plasmablasts, CXCL13 turned out to be the most predictable chemokine.

## Discussion

In order to further understand mechanisms that influence B cell trafficking into the CNS, we studied CSF levels of the B cell attracting chemokines CCL19, CCL21, CXCL12, CXCL13 and cytokines BAFF and APRIL. These findings were compared to baseline CSF parameters and detailed immune cell phenotyping of CSF cells in different groups of patients with neurological diseases. In contrast to previous studies, which had already shown an impact of different cytokines/chemokines on the fraction of B cells in the CSF compartment, we aimed to investigate the full repertoire of relevant chemokines/cytokines to understand the contribution and importance of each of them for humoral immune responses across different neuroinflammatory diseases.

CXCL13 turned out to show the strongest association with neuroinflammatory diseases. CXCL13 values were significantly elevated in patients with CIS, MS, LNB and OIND when compared to patients with NIND. Patients with OIND and LNB displayed highest values. However, the presence of very high CXCL13 levels in CSF is not specific for LNB as suggested previously [11,15-17]. Also CXCL12 and BAFF showed an association with neuroinflammation in patients with LNB and OIND. We observed highest CXCL12 CSF concentrations in patients with LNB and a subgroup of patients with OIND. Elevated BAFF levels were observed in patients with LNB and OIND compared to patients with NIND, CIS and MS. CSF levels of APRIL were slightly higher in patients with MS, Lyme disease and OIND. CCL19 CSF levels did not differ significantly among patients.

We observed a strong correlation between cytokine/chemokine levels in the CSF and other humoral and cellular CSF parameters. CD19^+^ B cells significantly correlated with CXCL13, CXCL12 and BAFF levels, with CXCL13 showing the strongest correlation. All chemokine and cytokine concentrations examined in this study significantly correlated with CD19^+^CD138^+^ plasmablasts in the CSF, again CXCL13 showed the lowest *P*- and highest r values. The impact of CXCL13 on the local humoral immune response was further strengthened by a strong correlation with intrathecal IgG, IgA and IgM synthesis and oligoclonal bands. Although IgA synthesis additionally correlated with APRIL, BAFF and CXCL12 and IgM synthesis with APRIL and BAFF, the correlation of CXCL13 showed lowest *P*- and highest r values. Similar findings were obtained when a subgroup of patients with an intact blood CSF barrier was examined.

In particular, the multiple regression analysis suggests that CXCL13 is the most important chemokine for the recruitment of B cells to the CNS compartment. CXCL13 and, to a lesser extent, BAFF seem to play a major role in the recruitment of plasmablasts. The source of the cytokines found in the CSF is uncertain. They might be produced locally in the CNS or originate from the periphery, especially in patients with a disrupted blood-CSF barrier. Given the high levels of some chemokines/cytokines in the CSF, which often exceed serum levels (for example, CXCL13) and the low transfer rate of proteins across the blood-CSF barrier (<1%) [30], it is conceivable to assume that at least in patients with an intact barrier most of the cytokines/chemokines found in the CSF are produced within the CNS compartment.

Furthermore, the significant correlation of cytokines/chemokines between each other in patients with a disrupted blood-CSF barrier suggests that the different cytokines/chemokines are regulated in parallel in patients with neuroinflammation. By means of stepwise multiple regression, we could further evaluate the effects among the different cytokines/chemokines and again point out the major role of CXCL13 for the presence of B cells and plasmablasts in the CSF.

## Conclusions

Our findings are consistent with previous studies, which suggested CXCL13 to be an essential chemokine in controlling the recruitment of B cells to the CNS in specific inflammatory diseases like multiple sclerosis [14] and Lyme neuroborreliosis [11]. However, none of the cytokines/chemokines that we studied is specific for a defined disease entity as suggested previously [11,15-17]. Taken together, our study provides a detailed analysis on cytokines/chemokines in the CSF of patients with a broad range of different neuroinflammatory diseases and points out the role of CXCL13 as the major chemoattractant for B cells. The results are helpful for future studies, which will address the kinetics of these cytokines during the course of disease and its relation to particular disease endophenotypes.

## Abbreviations

APRIL: A proliferation inducing ligand; BAFF: B cell activating factor; BCA-1: B cell attracting chemokine-1; BCMA: B cell maturation; CCL19: Chemokine (C-C motif) ligand 19; CCL21: Chemokine (C-C motif) ligand 21; CCR-7: Chemokine (C-C motif) receptor 7; CIS: Clinically isolated syndrome; CNS: Central nervous system; CSF: Cerebrospinal fluid; CXCL12: Chemokine (C-X-C motif) ligand 12; CXCL13: Chemokine (C-X-C motif) ligand 13; CXCR4: Chemokine (C-X-C motif) receptor 4; CXCR5: Chemokine (C-X-C motif) receptor 5; HIV: Human immunodeficiency virus; IgG: Immunoglobulin G; IgA: Immunoglobulin A; IgM: Immunoglobulin M; LNB: Lyme neuroborreliosis; MIP-3β: Macrophage inflammatory protein-3; MS: Multiple sclerosis; NIND: Non-inflammatory neurological diseases; OCBs: Oligoclonal bands; OIND: Other inflammatory neurological diseases; SDF-1: Stromal cell-derived factor-1; TACI: Transmembrane activator and CAML interacting protein; TNF: Tumor necrosis factor; SLC: Secondary lymphoid-tissue chemokine.

## Competing interests

There are no competing financial or non-financial interests by any of the authors.

## Authors’ contributions

MK (first author) carried out ELISA tests, all analyses, statistical analysis and interpretation of data, and wrote the first draft of the manuscript. SC took part in sample acquisition, conception and design. JS participated in interpretation of data and correction of the manuscript. VG carried out nephelometry and flow cytometry. MW interpreted data and corrected the manuscript. TK and AB were responsible for study conception, interpretation of data and correction of the manuscript. BH (corresponding author) was responsible for study conception, interpretation of data, correction of the manuscript, and final approval of the version to be published. All authors read and approved the final manuscript.

## Supplementary Material

Additional file 1 **Figure S1.** Chemokine/Cytokine levels in serum. CXCL13, CXCL12, CCL19, CCL21 and BAFF and APRIL serum levels of patients with NIND, CIS, MS, LNB and OIND are shown. Significant *P-*values for the comparison between groups are displayed. Click here for file

Additional file 2 **Table S1 **Correlation of CSF cytokine/chemokine levels and CSF parameters or percentage of immune cells. Samples with an intact blood-CSF barrier. Click here for file

Additional file 3 **Table S2 **Correlation of CSF cytokine/chemokine levels and CSF parameters or absolute count of immune cells. All samples. Click here for file
