# Supplementary figures and images for: CXCL13 is the major determinant for B cell recruitment to the CSF during neuroinflammation
Source: J Neuroinflammation. 2012 May 16;9:93. doi: 10.1186/1742-2094-9-93 (PMC3418196; doi:10.1186/1742-2094-9-93)

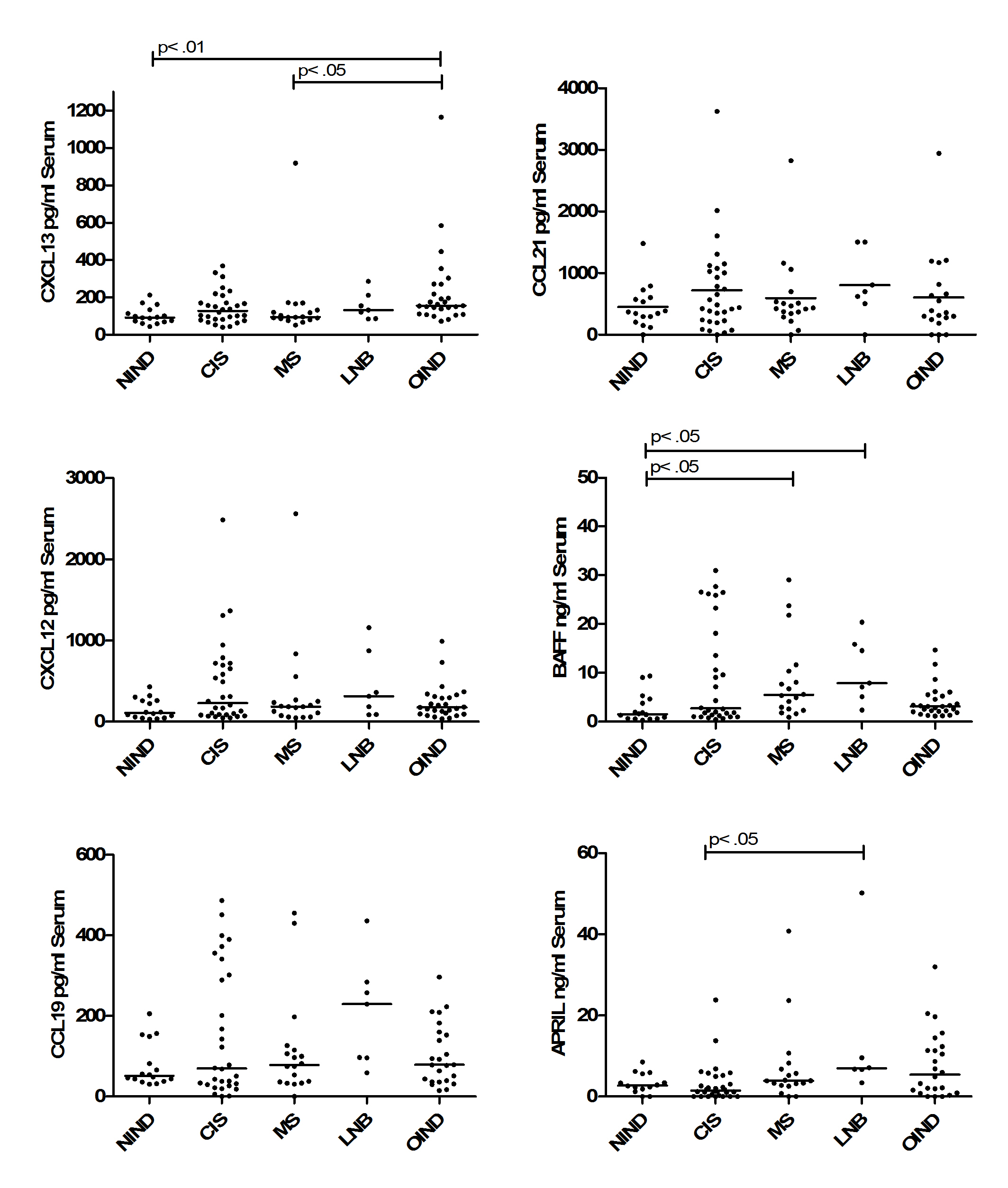

Supplement: Additional file 1 — Figure S1. Chemokine/Cytokine levels in serum. CXCL13, CXCL12, CCL19, CCL21 and BAFF and APRIL serum levels of patients with NIND, CIS, MS, LNB and OIND are shown. Significant P-values for the comparison between groups are displayed. [file 1742-2094-9-93-S1.jpeg]
