# Supplementary material for: CXCL13 is the major determinant for B cell recruitment to the CSF during neuroinflammation
Source: J Neuroinflammation. 2012 May 16;9:93. doi: 10.1186/1742-2094-9-93 (PMC3418196; doi:10.1186/1742-2094-9-93)
Supplement: Additional file 2 — Table S1 Correlation of CSF cytokine/chemokine levels and CSF parameters or percentage of immune cells. Samples with an intact blood-CSF barrier. [file 1742-2094-9-93-S2.doc]

Table S1: Correlation of CSF cytokine/chemokine levels and CSF parameters or percentage of immune cells. Samples with an intact blood-CSF barrier.

|  | APRIL | BAFF | CXCL12 | CXCL13 | CCL19 |
| --- | --- | --- | --- | --- | --- |
| Cell count | p = 0.6510  r = 0.05811 | p = 0.8080  r = 0.03098 | ***p = 0.0057***  ***r = 0.3419*** | ***p < 0.0001***  ***r = 0.5978*** | ***p = 0.0002***  ***r = 0.4485*** |
| Q_alb_ | p = 0.7382  r = -0.04295 | p = 0.1531  r = -0.1807 | ***p = 0.0162***  ***r = 0.2995*** | p = 0.8264  r = 0.02796 | p = 0.0616  r = 0.2369 |
| Intrathecal IgG | p = 0.5898  r = 0.06923 | ***p = 0.0456***  ***r = 0.2508*** | p = 0.4726  r = 0.09139 | ***p < 0.0001***  ***r = 0.5445*** | ***p = 0.0084***  ***r = 0.3292*** |
| Intrathecal IgA | p = 0.1762  r = 0.1726 | p = 0.3639  r = 0.1154 | p = 0.1181  r = 0.1973 | ***p = 0.0130***  ***r = 0.3089*** | p = 0.8649  r = 0.02187 |
| Intrathecal IgM | ***p = 0.0362***  ***r = 0.2644*** | p = 0.8951  r = -0.01680 | p = 0.4520  r = 0.09567 | ***p = 0.0109***  ***r = 0.3164*** | p = 0.3333  r = 0.1239 |
| OCBs | p = 0.4798  r = -0.09463 | p = 0.5920  r = -0.07121 | p = 0.3382  r = -0.1269 | ***p = 0.0109***  ***r = 0.3164*** | p = 0.0916  r = 0.2236 |
| All B cells  (CD19^+^) | p = 0.9639  r = -0.0058 | p = 0.9685  r = -0.005082 | p = 0.0666  r = 0.2326 | ***p < 0.0001***  ***r = 0.6660*** | ***p = 0.0078***  ***r = 0.3350*** |
| B cells  (CD19^+^D138^-^) | p = 0.9955  r = 0.0007 | p = 0.9135  r = 0.0139 | p = 0.0711  r = 0.2290 | ***p < 0.0001***  ***r = 0.6889*** | ***p = 0.0174***  ***r = 0.3012*** |
| Plasmablasts  (CD19^+^CD138^+^) | p = 0.1734  r = 0.1751 | p = 0.2043  r = 0.1621 | p = 0.0946  r = 0.2124 | ***p < 0.0001***  ***r = 0.5818*** | ***p = 0.0004***  ***r = 0.4335*** |
| T cells  (CD3^+^) | p = 0.5063  r = 0.08673 | p = 0.4618  r = -0.09518 | p = 0.0616  r = -0.2388 | p = 0.7581  r = 0.03991 | p = 0.2696  r = -0.1436 |
| Monocytes  (CD14^+^) | p = 0.4324  r = -0.1024 | p = 0.3145  r = -0.1298 | p = 0.4160  r = -0.1052 | ***p < 0.0001***  ***r = -0.5980*** | ***p = 0.0054***  ***r = -0.3517*** |

Spearman test was applied to correlate CSF cytokine/chemokine levels with CSF parameters or percentage of immune cells. Only samples with an intact blood-CSF barrier were included in this analysis, significant values are displayed in bold. Abbreviations: cerebrospinal fluid (CSF), albumin quotient (Q_alb_), oligoclonal bands (OCBs).
