# Supplementary material for: CXCL13 is the major determinant for B cell recruitment to the CSF during neuroinflammation
Source: J Neuroinflammation. 2012 May 16;9:93. doi: 10.1186/1742-2094-9-93 (PMC3418196; doi:10.1186/1742-2094-9-93)
Supplement: Additional file 3 — Table S2 Correlation of CSF cytokine/chemokine levels and CSF parameters or absolute count of immune cells. All samples. [file 1742-2094-9-93-S3.doc]

Table S2: Correlation of CSF cytokine/chemokine levels and CSF parameters or absolute count of immune cells. All samples.

|  | APRIL | BAFF | CXCL12 | CXCL13 | CCL19 |
| --- | --- | --- | --- | --- | --- |
| Cell count | ***p < 0.0001***  ***r = 0.4350*** | ***p < 0.0001***  ***r = 0.4967*** | ***p < 0.0001***  ***r = 0.4954*** | ***p < 0.0001***  ***r = 0.7583*** | ***p < 0.0001***  ***r = 0.5461*** |
| All B cells  (CD19^+^) | ***p < 0.0001***  ***r = 0.3916*** | ***p < 0.0001***  ***r = 0.4935*** | ***p < 0.0001***  ***r = 0.4840*** | ***p < 0.0001***  ***r = 0.7959*** | ***p < 0.0001***  ***r = 0.4700*** |
| B cells  (CD19^+^D138^-^) | ***p < 0.0001***  ***r = 0.3734*** | ***p < 0.0001***  ***r = 0.5062*** | ***p < 0.0001***  ***r = 0.4791*** | ***p < 0.0001***  ***r = 0.7978*** | ***p < 0.0001***  ***r = 0.4504*** |
| Plasmablasts  (CD19^+^CD138^+^) | ***p = 0.0002***  ***r = 0.3515*** | ***p < 0.0001***  ***r = 0.4092*** | ***p < 0.0001***  ***r = 0.3907*** | ***p < 0.0001***  ***r = 0.7512*** | ***p < 0.0001***  ***r = 0.4447*** |
| T cells  (CD3^+^) | ***p < 0.0001***  ***r = -0.4466*** | ***p < 0.0001***  ***r = 0.4631*** | ***p < 0.0001***  ***r = 0.4748*** | ***p < 0.0001***  ***r = 0.6030*** | ***p < 0.0001***  ***r = 0.4688*** |
| Monocytes  (CD14^+^) | ***p = 0.0338***  ***r = 0.2044*** | ***p = 0.0237***  ***r = 0.2176*** | ***p = 0.0022***  ***r = 0.2920*** | ***p = 0.0206***  ***r = 0.2226*** | ***p = 0.0012***  ***r = 0.3067*** |

Spearman test was applied to correlate CSF cytokine/chemokine levels with CSF absolute count of immune cell subsets. All samples (with and without an intact blood-CSF barrier) were included in the analysis, significant values are displayed in bold. Abbreviations: cerebrospinal fluid (CSF).
